# Supplementary material for: Food Sources of Shortfall Nutrients among Latin Americans: Results from the Latin American Study of Health and Nutrition (ELANS)
Source: Int J Environ Res Public Health. 2021 May 7;18(9):4967. doi: 10.3390/ijerph18094967 (PMC8125540; doi:10.3390/ijerph18094967)
Supplement: Supplementary file 1 [file ijerph-18-04967-s001.zip › ijerph-1187828-supplementary.pdf]

Supplement table 1 – Shortfall nutrients prevalence of inadequacy for the overall sample and each of the eight Latin American countries. Latin American Study of Nutrition and Health/ *Estudio Latinoamericano de Nutrición y Salud* (ELANS) – 2015.

	Vitamin A (μg/day)	Vitamin C (mg/day)	Vitamin D (μg/day)	Vitamin E (mg/day)	Calcium (mg/day)	Magnesium (mg/day)	Potassium (mg/day)[1]	Fiber (g/day)[1]	Iron (mg/day)
**Overall ELANS sample (n= 9218)**									
**Male**									
15-18y	53.6	39.7	99.7	100.0	95.4	88.5	0.2	0.0	4.0
19-50y	50.0	38.2	99.5	100.0	77.3	78.8	5.8	3.6	1.7
51-65y	51.6	39.4	99.6	100.0	77.6	89.1	6.4	7.2	2.2
**Female**									
15-18y	39.0	30.5	99.8	100.0	99.0	87.3	10.4	0.0	13.3
19-50y	39.4	35.6	99.8	100.0	86.0	69.5	27.0	3.1	20.7
51-65y	39.4	35.6	99.9	100.0	98.0	75.5	28.3	6.9	4.2
**Argentina (n= 1266)**									
**Male**									
15-18y	59.5	39.4	100.0	99.8	85.3	98.7	1.3	0.0	0.4
19-50y	57.1	37.8	100.0	100.0	52.8	97.1	5.4	4.1	0.4
51-65y	59.9	34.8	100.0	100.0	52.8	98.7	5.5	10.1	0.1
**Female**									
15-18y	48.4	33.0	100.0	100.0	94.2	99.7	15.9	0.0	12.1
19-50y	49.2	35.6	100.0	100.0	69.2	92.9	30.3	3.2	15.0
51-65y	50.8	34.5	100.0	100.0	94.2	92.1	33.3	8.2	0.5
**Brazil (n= 2000)**									
**Male**									
15-18y	59.5	39.4	100.0	99.4	98.3	90.3	0.0	0.0	13.6
19-50y	57.1	37.8	100.0	99.9	93.3	87.3	7.4	2.5	5.6
51-65y	59.9	34.8	100.0	99.4	96.0	97.4	7.2	7.9	9.2
**Female**									
15-18y	48.4	33.0	100.0	100.0	100.0	96.2	12.1	0.0	30.1
19-50y	49.2	35.6	100.0	100.0	96.2	89.2	30.5	5.9	43.3
51-65y	50.8	34.5	100.0	100.0	99.9	89.6	28.1	8.0	14.8
**Chile (n=879)**									

**Male**									
15-18y	80.0	55.2	99.8	100.0	97.2	99.3	0.0	0.0	5.1
19-50y	71.6	64.4	100.0	100.0	83.9	98.0	9.4	5.9	1.0
51-65y	77.6	67.7	100.0	100.0	91.6	100.0	8.0	8.0	0.5
**Female**									
15-18y	41.3	40.1	99.9	100.0	99.5	99.3	5.1	0.0	16.5
19-50y	50.4	50.0	100.0	100.0	95.0	97.8	27.7	2.9	23.0
51-65y	49.6	51.6	100.0	100.0	99.7	99.6	23.8	4.0	1.7
**Colombia (n=1230)**									
**Male**									
15-18y	32.6	17.6	99.6	100.0	93.2	76.4	0.0	0.0	0.5
19-50y	32.3	23.6	99.5	99.3	64.4	66.6	5.3	3.9	0.0
51-65y	35.9	28.1	97.9	99.9	57.5	67.4	5.3	3.0	0.1
**Female**									
15-18y	24.2	17.4	99.7	100.0	93.7	62.6	5.9	0.0	2.6
19-50y	22.1	20.1	99.5	99.9	70.2	42.9	20.3	3.2	8.5
51-65y	23.6	18.9	99.9	99.9	95.5	53.2	22.2	6.3	0.1
**Costa Rica (n=798)**									
**Male**									
15-18y	46.4	42.5	100.0	100.0	99.8	88.9	0.0	0.0	0.4
19-50y	38.6	47.2	100.0	100.0	94.7	67.4	5.1	3.4	0.2
51-65y	44.8	49.2	100.0	100.0	98.6	88.9	1.9	5.8	2.5
**Female**									
15-18y	40.1	44.0	100.0	98.7	100.0	94.3	7.9	0.0	14.4
19-50y	31.2	38.6	100.0	100.0	98.5	73.2	27.8	5.1	16.9
51-65y	31.2	37.8	100.0	100.0	100.0	83.9	32.6	6.7	0.9
**Ecuador (n=800)**									
**Male**									
15-18y	54.4	12.7	94.6	98.5	96.1	89.4	0.0	0.0	0.5
19-50y	45.6	11.9	84.6	97.5	64.8	59.1	4.4	3.7	0.0
51-65y	51.2	18.1	82.1	99.9	76.4	80.5	8.1	8.2	1.5
**Female**									
15-18y	34.8	11.7	91.6.	98.7	99.9	76.7	13.6	0.0	7.0

19-50y	37.5	10.2	89.4	99.4	79.4	40.9	25.8	1.4	10.8
51-65y	37.5	11.1	86.7	99.6	95.1	48.4	26.4	1.4	0.3
**Peru (n=1113)**									
**Male**									
15-18y	44.8	21.8	96.1	100.0	100.0	80.5	0.0	0.0	0.6
19-50y	37.5	26.8	95.8	100.0	98.2	66.3	5.2	4.3	0.1
51-65y	46.0	32.3	97.7	100.0	99.7	89.8	9.3	6.7	0.0
**Female**									
15-18y	21.5	28.1	99.7	100.0	100.0	83.9	11.5	0.0	8.1
19-50y	26.1	23.6	97.4	100.0	99.4	50.8	26.8	2.6	11.6
51-65y	24.2	22.4	99.9	100.0	100.0	57.5	30.1	6.8	0.1
**Venezuela (1132)**									
**Male**									
15-18y	62.2	23.3	100.0	100.0	88.5	82.4	0.0	0.0	2.4
19-50y	58.3	24.5	100.0	100.0	62.9	78.8	3.7	2.3	0.3
51-65y	73.2	25.5	100.0	100.0	72.2	90.7	5.3	9.3	0.8
**Female**									
15-18y	57.5	14.0	100.0	100.0	96.9	88.1	9.5	0.0	10.2
19-50y	52.8	17.4	100.0	100.0	77.6	64.1	24.1	2.6	17.4
51-65y	46.0	15.9	100.0	100.0	95.5	60.6	30.0	10.9	1.8

All nutrients % of inadequacy was evaluated according to EAR (Estimated Average Requirements), with exception for [1]fiber and potassium which were calculated the % of recommendation AI (adequate intake)

Percentage of inadequacy was not calculated for folate given the inconsistencies in standardization of this nutrient in the ELANS sample.

Supplement Table 2 – Food sources of vitamin A RAE (μg/day) intake among Latin American adolescents and adults. The Latin American Study of Nutrition and Health.

Main Group	Subgroup	Categories	Cons	Mean± SE	%
		**Argentina (n=1266)**			
Protein Foods	Meats	Liver and organ meats	75	3269.3±702.3	12.1
Vegetables	Vegetables, excluding potatoes	Carrots	246	884.5±51.5	11.1
Vegetables	Vegetables, excluding potatoes	Other Vegetables and Combinations	825	261.6±10.8	9.5
Mixed Dishes	Mixed Dishes – Grain-based	Pasta Mixed Dishes	794	245.3±11.8	9.0
Mixed Dishes	Mixed Dishes – Pizza	Pizza	372	422.7±15.4	7.8
		**Brazil (n=2000)**			
Protein Foods	Meats	Liver and organ meats	107	111011.1±893.2	31.9
Vegetables	Vegetables, excluding potatoes	Other Starchy Vegetables	200	1571.9±220.9	8.5
Vegetables	Vegetables, excluding potatoes	Lettuces and Lettuces Salads	1067	260.4±6.5	7.5
Fats and Oils	Fats and Oils	Margarine	1672	160.2±4.2	7.3
Vegetables	Vegetables, excluding potatoes	Carrots	371	607.9±30.5	6.1
		**Chile (n=879)**			
Vegetables	Vegetables, excluding potatoes	Lettuce and lettuce salads	637	239.8±8.0	16.9
Condiments and sauces	Condiments and Sauces	Dips, gravies, other sauces	109	544.2±56.8	6.6
Protein Foods	Eggs	Eggs and omelets	488	121.2±3.7	6.6
Vegetables	Vegetables, excluding potatoes	Carrots	191	305.5±14.4	6.5
Milk and Dairy	Cheese	Cheese	656	77.7±2.8	5.7
		**Colombia (n=)**			
Protein Foods	Meats	Liver and organ meats	101	3274.6±584.8	16.9
Mixed Dishes	Mixed Dishes – M/P/S	Meat mixed dishes	493	589.9±30.3	14.7
Protein Foods	Eggs	Eggs and Omelets	1506	133.9±1.9	10.3
Milk and Dairy	Milk	Milk, whole	1514	91.9±1.5	7.1
Mixed Dishes	Mixed Dishes - Soups	Soups	902	128.7±7.6	5.7
		**Costa Rica (n=)**			
Protein Foods	Meats	Liver and organ meats	38	3860.7±569.1	12.8
Snacks and Sweets	Sweet Bakery Products	Doughnuts, sweet rolls, pastries	219	665.2±65.7	12.8
Sugars	Sugars	Sugars and Honey	1678	65.7±1.7	9.7
Beverages	100% Juices	Vegetable Juice	8	1372.2±169.8	6.7

Milk and Dairy	Milk	Milk, reduced fat	477	123.3±5.3	5.2
**Ecuador (n=)**					
Protein Foods	Meats	Liver and Organ Meats	51	6431.8±1045.7	28.6
Mixed Dishes	Mixed Dishes – Soups	Soups	1459	81.1±2.7	8.9
Mixed Dishes	Mixed Dishes – M/P/S	Meat Mixed Dishes	362	275.5±11.4	7.9
Vegetables	Vegetables, excluding potatoes	Carrots	283	237.1±14.2	5.8
Milk and Dairy	Cheese	Cheese	634	98.6±2.9	5.4
**Peru**					
Protein Foods	Meats	Liver and organ meats	225	1506.7±166.3	23.8
Vegetables	Vegetables, excluding potatoes	Other starchy vegetables	439	408.8±36.1	12.6
Mixed Dishes	Mixed Dishes – Soups	Soups	1214	120.0±3.8	8.8
Protein Foods	Eggs	Eggs and Omelets	1028	83.7±1.6	6.1
Mixed Dishes	Mixed Dishes - Grains	Rice Mixed Dishes	541	134.0±5.0	4.8
**Venezuela (n=)**					
Protein Foods	Meats	Liver and organ meats	38	10862.4±1571.3	20.2
Beverages	100% Juices	Other Fruit Juice	1013	175.1±8.3	8.9
Vegetables	Vegetables, excluding potatoes	Carrots	220	651.8±30.8	7.9
Milk and Dairy	Cheese	Cheese	1932	71.0±1.0	7.5
Fats and Oils	Fats and Oils	Margarine	1069	91.01±1.6	5.3

Note: M/P/S: Meat, Poultry, Seafood; SE: standard error

Supplement table 3 – Food sources of vitamin D (mg/day) intake among Latin American adolescents and adults. The Latin American Study of Nutrition and Health.

Main Group	Subgroup	Categories	Cons	Mean± SE	%
**Argentina (n=1226)**					
Milk and Dairy	Milk	Milk, whole	545	2.25±0.08	14.5
Protein Foods	Eggs	Eggs and Omelets	605	1.33±0.04	9.1
Mixed Dishes	Mixed Dishes - Grains	Pasta Mixed Dishes	794	1.03±0.04	8.8
Milk and Dairy	Milk	Milk, reduced fat	290	2.18±0.07	7.5
Protein Foods	Seafood	Fish	126	5.22±0.73	7.2
**Brazil (n=2000)**					

Milk and Dairy	Milk	Milk, whole	2382	2.12±0.03	38.8
Protein Foods	Seafood	Fish	370	4.18±0.35	11.9
Protein Foods	Meats	Beef, excludes ground	1882	0.62±0.01	9.0
Protein Foods	Eggs	Eggs and Omelets	599	1.53±0.03	7.0
Milk and Dairy	Cheese	Cheese	839	0.53±0.06	3.4
**Chile (n=879)**					
Protein Foods	Seafood	Fish	1029	1.24±0.16	22.6
Protein Foods	Eggs	Eggs and Omelets	488	1.60±0.04	13.9
Milk and Dairy	Milk	Milk, whole	208	3.20±0.13	11.8
Milk and Dairy	Milk	Milk, reduced fat	191	3.24±0.10	11.0
Protein Foods	Meats	Beef, excludes ground	393	0.89±0.04	6.2
**Colombia (n=1230)**					
Milk and Dairy	Milk	Milk, whole	1514	2.54±0.04	29.8
Protein Foods	Eggs	Eggs and Omelets	1506	1.63±0.02	18.9
Protein Foods	Seafood	Fish	279	4.27±0.30	9.23
Milk and Dairy	Flavored Milk	Flavored Milk, whole	652	1.56±0.08	7.9
Beverages	Coffee and Tea	Coffee	2523	0.24±0.02	4.6
**Costa Rica (n=798)**					
Milk and Dairy	Milk	Milk, reduced fat	477	2.12±0.09	19.9
Protein Foods	Eggs	Eggs and Omelets	588	1.31±0.03	15.2
Protein Foods	Seafoods	Fish	285	2.53±0.25	14.2
Milk and Dairy	Cheese	Cheese	445	0.80±0.04	6.9
Protein Foods	Meats	Pork	212	0.80±0.04	6.9
**Ecuador (n=800)**					
Protein Foods	Seafoods	Fish	350	8.69±0.49	30.8
Mixed Dishes	Mixed Dishes – M/P/S	Seafood Mixed Dishes	278	6.81±0.49	16.2
Milk and Dairy	Milk	Milk, whole	525	2.88±0.05	15.4
Protein Foods	Eggs	Eggs and Omelets	636	1.28±0.03	8.13
Milk and Dairy	Cheese	Cheese	634	1.17±0.04	7.6
**Peru (n=1113)**					
Protein Foods	Seafoods	Fish	528	10.91±0.49	45.5
Mixed Dishes	Mixed Dishes – M/P/S	Seafood Mixed Dishes	311	6.32±0.60	12.5
Protein Foods	Eggs	Eggs and Omelets	1028	1.22±0.02	9.7

Milk and Dairy	Milk	Milk, whole	564	1.42±0.06	8.3
Milk and Dairy	Yogurt	Yogurt, regular	144	2.25±0.12	2.6
		**Venezuela (n=1132)**			
Protein Foods	Eggs	Eggs and Omelets	727	1.39±0.03	14.3
Protein Foods	Seafood	Fish	297	3.19±0.26	13.3
Milk and Dairy	Milk	Milk, whole	254	2.94±0.13	11.4
Beverages	Coffee and Tea	Coffee	1821	0.31±0.01	8.7
Milk and Dairy	Cheese	Cheese	1932	0.29±0.01	8.7
Note: M/P/S: Meat/Poultry/Seafood; SE: standard error					

Supplement Table 4 – Food sources of vitamin E (mg/day) intake among Latin American adolescents and adults. The Latin American Study of Nutrition and Health.

Main group	Subgroup	Category	Cons	M± SE	%
		**Argentina (n=1266)**			
Beverages	Coffee and Tea	Tea	3897	10.43±0.16	62.8
Fats and Oils	Fats and Oils	Salad Dressings and Vegetables Oils	1057	3.75±0.10	6.1
Mixed Dishes	Mixed Dishes – M/P/S	Meat Mixed Dishes	781	4.90±0.16	5.3
Mixed Dishes	Mixed Dishes – Grains	Pasta Mixed Dishes	794	4.45±0.19	4.9
Mixed Dishes	Mixed Dishes – M/P/S	Poultry Mixed Dishes	383	5.94±0.21	3.2
		**Brazil (n=2000)**			
Protein Foods	Plant-Based Proteins	Beans, peas, legumes	3701	0.54±0.01	9.5
Fats and Oils	Fats and Oils	Margarine	1672	1.29±0.03	8.5
Grains	Cooked Grains	Rice	4680	0.28±0.00	6.2
Protein Foods	Meats	Beef, excludes ground	1882	0.64±0.01	5.7
Beverages	Coffee and Tea	Tea	183	5.19±0.65	4.5
		**Chile (n=879)**			
Fats and Oils	Fats and Oils	Salad Dressing and Vegetable Oils	1547	1.12±0.04	15.7
Fruits	Fruits	Other Fruit and Fruit Salads	751	0.94±0.03	6.4
Protein Foods	Seafoods	Fish	1029	0.65±0.04	6.1
Mixed Dishes	Mixed Dishes – Grains	Turnovers and other grain-based items	101	5.67±0.35	5.2
Protein Foods	Eggs	Eggs and Omelets	488	1.15±0.06	5.1

**Colombia (n=1230)**					
Protein Foods	Eggs	Eggs and Omelets	1506	0.92±0.01	7.8
Mixed Dishes	Meat Dishes – M/P/S	Meat Mixed Dishes	493	2.51±0.10	6.9
Beverages	Sweetened Beverages	Fruit Drinks	1116	0.92±0.04	5.8
Fruits	Fruits	Banana	1421	0.67±0.05	5.4
Snacks and Sweets	Savory Snacks	Potato Chips	189	5.00±0.37	5.2
**Costa Rica (n=798)**					
Grains	Cooked Grains	Rice	1845	1.66±0.02	24.8
Fats and Oils	Fats and Oils	Margarine	553	1.28±0.09	5.7
Protein Foods	Eggs	Eggs and Omelets	588	1.03±0.03	4.9
Mixed Dishes	Mixed Dishes - Mexican	Other Mexican Mixed Dishes	498	1.08±0.04	4.3
Protein Foods	Seafood	Fish	285	1.78±0.09	4.1
**Ecuador (n=800)**					
Grains	Cooked Grains	Rice	2408	1.63±0.03	21.3
Protein Foods	Eggs	Eggs and Omelets	636	3.53±0.14	9.3
Mixed Dishes	Mixed Dishes – Grains	Turnovers and other grain-based items	140	10.27±0.68	7.5
Mixed Dishes	Mixed Dishes - Soups	Soups	1459	1.06±0.03	7.3
Fruits	Fruits	Banana	583	1.99±0.18	6.29
**Peru (n=1113)**					
Fruits	Fruits	Other Fruit and Fruit Salads	991	1.57±0.12	11.4
Protein Foods	Eggs	Eggs and Omelets	1028	0.97±0.02	7.3
Grains	Cooked Grains	Rice	2574	0.33±0.00	6.3
Mixed Dishes	Mixed Dishes – Grains	Rice Mixed Dishes	541	1.63±0.04	6.0
Protein Foods	Seafoods	Fish	528	1.43±0.06	5.6
**Venezuela (n=1132)**					
Fats and Oils	Fats and Oils	Margarine	1069	1.38±0.02	11.7
Mixed Dishes	Mixed Dishes - Grains	Turnovers and other grain-based items	546	2.43±0.06	10.4
Mixed Dishes	Mixed Dishes – M/P/S	Meat Mixed Dishes	507	1.64±0.07	5.8
Protein Foods	Eggs	Eggs and Omelets	727	0.85±0.02	4.5
Grains	Quick breads, Bread Products	Pancakes, waffles, French Toast	2134	0.23±0.02	3.8

Note: M/P/S: Meat/Poultry/Seafood; SE: standard error

Supplement table 5 – Food sources of vitamin C (mg/day) intake among Latin American adolescents and adults. The Latin American Study of Nutrition and Health.

Main group	Subgroup	Category	Cons	M± SE	%
		**Argentina (n=1266)**			
Fruits	Fruits	Other Fruit and Fruit Salads	554	51.23±2.35	22.8
Beverages	Sweetened Beverages	Fruit Drinks	1450	12.32±1.15	14.4
Beverages	100% Juices	Citrus Juice	302	39.13±3.69	9.5
Vegetables	Vegetables, excluding potatoes	Tomatoes	659	12.90±0.73	6.9
Vegetables	Vegetables, excluding potatoes	Other Vegetables and Combinations	825	9.08±0.40	5.2
		**Brazil (n=2000)**			
Beverages	100% Juices	Other Fruit Juice	839	272.48±20.88	48.7
Fruits	Fruits	Other Fruit and Fruit Salads	1056	70.84±3.48	15.9
Beverages	100% Juices	Citrus Juice	702	95.32±3.59	14.2
Vegetables	Vegetables, excluding potatoes	Tomatoes	1120	13.25±0.66	3.2
Beverages	Sweetened Beverages	Fruit Drinks	1265	10.69±1.63	2.9
		**Chile (n=879)**			
Beverages	Sweetened Beverages	Fruit Drinks	757	30.97±1.85	22.1
Fruits	Fruits	Other Fruit and Fruit Salads	751	25.44±1.63	18.0
Vegetables	Vegetables, excluding potatoes	Tomatoes	768	12.10±0.23	8.8
Vegetables	Vegetables, excluding potatoes	Other Vegetables and Combinations	424	17.87±1.13	7.2
Fruits	Fruits	Strawberries and Berries	112	63.10±4.76	6.7
		**Colombia (n=1230)**			
Beverages	Sweetened Beverages	Fruit Drinks	1116	47.30±1.05	18.0
Fruits	Fruits	Other Fruit and Fruit Salads	697	71.73±4.81	16.9
Beverages	100% Juices	Citrus Juice	513	65.27±2.97	11.4
Beverages	100% Juice	Other Fruit Juice	433	60.71±2.50	8.97
Fruits	Fruits	Banana	1421	13.13±0.31	6.37
		**Costa Rica (n=798)**			
Fruits	Fruits	Other Fruit and Fruit Salads	490	58.51±4.03	23.8
Beverages	100% Juices	Citrus Juice	365	43.95±3.16	13.3
Vegetables	Vegetables, excluding potatoes	Other Vegetables and Combinations	734	9.97±0.49	6.1
Vegetables	Vegetables, excluding potatoes	Tomatoes	380	15.88±1.15	5.0

Vegetables	Vegetables, excluding potatoes	Other Red and Orange Vegetables	562	10.41±1.11	4.9
		**Ecuador (n=800)**			
Beverages	100% Juices	Citrus Juice	763	73.22±3.14	24.4
Fruits	Fruits	Other Fruit and Fruit Salads	640	57.33±3.03	16.8
Mixed Dishes	Mixed Dishes – M/P/S	Meat Mixed Dishes	362	51.38±33.74	7.8
Mixed Dishes	Mixed Dishes - Soups	Soups	1459	12.47±0.30	7.3
Mixed Dishes	Mixed Dishes – M/P/S	Poultry Mixed Dishes	436	35.50±0.84	6.8
		**Peru (n=1113)**			
Beverages	100% Juices	Other Fruit Juice	895	101.24±13.48	29.3
Fruits	Fruits	Other Fruit and Fruit Salads	991	48.81±2.78	17.5
Mixed Dishes	Mixed Dishes – M/P/S	Poultry Mixed Dishes	937	15.55±0.61	5.2
Beverages	100% Juices	Citrus Juice	1245	8.85±0.77	3.9
Fruits	Fruits	Banana	853	12.21±0.30	3.9
		**Venezuela (n=1132)**			
Beverages	100% Juices	Other Fruit Juice	1013	88.21±2.61	31.9
Beverages	100% Juices	Citrus Juice	402	116.88±4.38	18.2
Fruits	Fruits	Other Fruit and Fruit Salads	332	71.05±4.58	9.1
Beverages	Sweetened Beverages	Fruit Drinks	243	51.88±3.40	4.9
Mixed Dishes	Mixed Dishes – M/P/S	Meat Mixed Dishes	507	23.03±1.83	3.9

Note: M/P/S: Meat/Poultry/Seafoods; SE: Standard Error

Supplement table 6 – Food sources of dietary folate DFE (mg/day) intake among Latin American adolescents and adults. The Latin American Study of Nutrition and Health.

Main group	Subgroup	Category	Cons	M± SE	%
		**Argentina (n=1266)**			
Grains	Bread, Rolls, Tortillas	Yeast Breads	2583	153.5±2.16	29.9
Grains	Cooked grains	Pasta, noodles, cooked grains	565	226.68±5.81	9.4
Mixed Dishes	Mixed Dishes – Pizza	Pizza	372	251.70±8.86	6.9
Mixed Dishes	Mixed Dishes – Grain-based	Pasta Mixed Dishes	794	124.61±3.42	6.8
Mixed Dishes	Mixed Dishes - Soups	Soups	505	140.25±5.37	4.9
		**Brazil (n=2000)**			

Grains	Cooked Grains	Yeast Breads	3465	143.97±1.37	27.2
Grains	Cooked grains	Rice	4680	45.36±0.48	11.6
Protein Foods	Plant-based Protein Foods	Beans, Peas, Legumes	3701	46.87±0.57	9.4
Grains	Cooked grains	Pasta, Noodles, Cooked Grains	594	181.04±5.87	5.85
Mixed Dishes	Mixed Dishes – Grain-based	Pasta Mixed Dishes	600	141.99±4.26	4.64
		**Chile (n=879)**			
Grains	Bread, Rolls, Tortillas	Yeast Breads	1880	126.60±1.59	31.7
Protein Foods	Seafoods	Fish	1029	102.77±2.41	14.1
Grains	Cooked grains	Pasta, Noodles, Cooked Grains	358	142.32±3.62	6.8
Fruits	Fruits	Other Fruit and Fruit Salads	751	49.41±1.26	4.9
Vegetables	Vegetables, excluding potatoes	Lettuce and Lettuce Salads	637	47.28±1.82	4.0
		**Colombia (n=1230)**			
Grains	Cooked grains	Rice	3214	54.74±0.48	1.5
Grains	Bread, Rolls, Tortillas	Yeast Breads	1418	123.52±2.81	1.5
Protein Foods	Plant-based Protein Foods	Beans, Peas, Legumes	865	193.65±6.69	1.4
Grains	Quick Breads and Bread Products	Pancakes, Waffles, French Toast	794	85.80±1.48	0.6
Grains	Bread, Rolls, Tortillas	Rolls and Buns	496	122.60±4.21	0.5
		**Costa Rica (n=798)**			
Protein Foods	Plant-based Protein Foods	Beans, Peas, Legumes	1213	132.55±2.73	21.9
Grains	Cooked grains	Rice	1845	44.21±0.55	11.2
Grains	Bread, Rolls, Tortillas	Yeast Breads	1138	69.75±1.46	10.9
Mixed Dishes	Mixed Dishes - Mexico	Other Mexican Mixed Dishes	498	133.69±3.55	9.1
Fruits	Fruits	Other Fruit and Fruit Salads	490	45.48±2.27	3.1
		**Ecuador (n=800)**			
Grains	Cooked grains	Rice	2408	71.63±0.86	21.9
Mixed Dishes	Mixed Dishes - Soups	Soups	1459	95.06±2.72	15.4
Grains	Bread, Rolls, Tortillas	Rolls and Buns	776	107.10±2.68	10.6
Grains	Bread, Rolls, Tortillas	Yeast Breads	446	86.63±3.66	4.9
Beverages	100% Juice	Citrus Juice	763	40.45±1.94	3.8
		**Peru (n=1113)**			
Grains	Bread, Rolls, Tortillas	Yeast Breads	2482	112.83±1.42	21.2
Grains	Cooked grains	Rice	2574	102.53±0.95	20.1
Mixed Dishes	Mixed Dishes - Soups	Soups	1214	97.60±2.92	7.5

Mixed Dishes	Mixed Dishes – Grain-based	Rice Mixed Dishes	541	185.26±4.57	7.0
Protein Foods	Plant-Based Protein Foods	Beans, Peas, Legumes	643	99.16±3.86	4.8
		**Venezuela (n=1132)**			
Grains	Quick Breads and Bread Products	Pancakes, Waffles, French Toast	2134	153.81±1.81	25.8
Grains	Bread, Rolls, Tortillas	Yeast Breads	958	184.27±3.76	13.9
Grains	Cooked grains	Pasta, Noodles, Cooked Grains	485	278.31±5.89	10.2
Grains	Cooked grains	Rice	1482	47.97±0.64	5.4
Mixed Dishes	Mixed Dishes – Grain-based	Turnovers and other grain-based items	546	101.14±3.53	4.3

Note: M/PS: Meat/Poultry/Seafood; SE: Standard Error

Supplement table 7– Food sources of calcium (mg/day) intake among Latin American adolescents and adults. The Latin American Study of Nutrition and Health.

Main group	Subgroup	Category	Cons	M± SE	%
		**Argentina (n=1266)**			
Mixed Dishes	Mixed Dishes – Pizza	Pizza	372	928.69±33.99	18.1
Milk and Dairy	Cheese	Cheese	1005	268.22±6.70	14.5
Grains	Bread, rolls, tortillas	Yeast Breads	2583	47.82±1.26	6.6
Mixed Dishes	Mixed Dishes – Grain-based	Pasta Mixed Dishes	794	150.96±6.50	5.9
Milk and Dairy	Milk	Milk, Whole	545	199.79±6.70	5.9
		**Brazil (n=2000)**			
Milk and Dairy	Milk	Milk, whole	2382	187.79±2.63	21.9
Milk and Dairy	Cheese	Cheese	839	249.89±9.07	10.3
Protein Foods	Plant-based Protein Foods	Beans, Peas, Legumes	3701	45.84±0.51	8.3
Grains	Bread, rolls, tortillas	Yeast Breads	3465	40.72±0.73	6.9
Milk and Dairy	Flavored Milk	Flavored Milk, whole	475	149.68±5.67	3.5
		**Chile (n=879)**			
Milk and Dairy	Cheese	Cheese	656	285.33±9.09	21.1
Grains	Bread, rolls, tortillas	Yeast Breads	1880	49.52±1.18	10.5
Milk and Dairy	Milk	Milk, reduced fat	191	325.71±9.53	7.0
Milk and Dairy	Milk	Milk, whole	208	286.42±12.22	6.7
Milk and Dairy	Yogurt	Yogurt, regular	240	180.75±4.06	4.9

		**Colombia (n=1230)**			
Milk and Dairy	Milk	Milk, whole	1514	229.49±3.73	17.9
Grains	Bread, rolls, tortillas	Yeast breads	1418	176.30±4.13	12.9
Milk and Dairy	Cheese	Cheese	568	279.23±8.18	8.2
Milk and Dairy	Flavored Milk	Flavored milk whole	652	221.35±8.15	7.5
Protein Foods	Eggs	Eggs and Omelets	1506	46.84±0.76	3.6
		**Costa Rica (n=798)**			
Grains	Bread, rolls, tortillas	Yeast breads	1138	141.19±3.35	18.8
Milk and Dairy	Milk	Milk, reduced fat	477	208.18±8.90	11.6
Milk and Dairy	Cheese	Cheese	445	169.95±8.33	8.9
Grains	Cooked Grains	Rice	1845	23.36±0.30	5.1
Protein Foods	Plant-based Protein Foods	Beans, peas, legumes	1213	29.93±0.66	4.3
		**Ecuador (n=800)**			
Milk and Dairy	Cheese	Cheese	634	252.00±7.33	14.8
Milk and Dairy	Milk	Milk, whole	525	259.02±4.73	12.6
Mixed Dishes	Mixed Dishes - Soup	Soups	1459	89.84±1.60	10.6
Grains	Bread, rolls, tortillas	Rolls and Buns	776	129.27±3.26	9.3
Grains	Bread, rolls, tortillas	Yeast breads	446	125.04±5.35	5.2
		**Peru (n=1113)**			
Milk and Dairy	Cheese	Cheese	557	248.04±9.48	11.1
Milk and Dairy	Milk	Milk, whole	564	232.48±7.17	10.6
Grains	Bread, rolls, tortillas	Yeast breads	2482	43.92±0.95	8.9
Sugars	Sugars	Sugars and honey	5192	13.91±0.16	5.9
Grains	Cooked grains	Rice	2574	25.50±0.24	5.4
		**Venezuela (n=1132)**			
Milk and Dairy	Cheese	Cheese	1932	290.07±3.98	34.7
Grains	Quick breads and bread products	Pancakes, Waffles, French Toast	2134	85.90±3.13	11.2
Mixed Dishes	Mixed Dishes – Grain-based	Turnovers and other grain-based items	546	271.50±16.46	9.1
Milk and Dairy	Milk	Milk, whole	254	260.21±11.13	4.1
Grains	Bread rolls, tortillas	Yeast breads	958	68.60±2.24	4.0

Note: M/P/S: Meat/Poultry/Seafood; SE: Standard Error

Supplement table 8 – Food sources of magnesium (mg/day) intake among Latin American adolescents and adults. The Latin American Study of Nutrition and Health.

Main group	Subgroup	Category	Cons	M± SE	%
		**Argentina (n=1266)**			
Grains	Bread, Rolls and Tortillas	Yeast Breads	2583	22.03±0.37	10.9
Mixed Dishes	Mixed Dishes – Grain-based	Pasta Mixed Dishes	794	45.47±1.52	6.3
Mixed Dishes	Mixed Dishes – M/P/S	Meat Mixed Dishes	781	37.87±0.82	5.1
Beverages	Tea	Tea	3897	6.53±0.07	4.9
Mixed Dishes	Mixed Dishes - Soup	Pizza	372	65.75±2.29	4.6
		**Brazil (n=2000)**			
Protein Foods	Plant-based Protein Foods	Beans, peas, legumes	3701	32.48±0.36	14.1
Grains	Bread, Rolls and Tortillas	Yeast breads	3465	19.07±0.18	7.7
Grains	Cooked grains	Rice	4680	12.39±0.12	6.8
Protein Foods	Meat	Beef, excludes ground	1882	27.20±0.41	6.0
Alcoholic Beverage	Alcoholic Beverage	Beer	444	101.92±5.17	5.3
		**Chile (n=879)**			
Grains	Bread, Rolls and Tortillas	Yeast breads	1880	26.38±0.35	14.9
Protein Foods	Seafoods	Fish	1029	25.22±0.49	7.8
Fruits	Fruits	Other Fruit and Fruit Salads	751	17.84±0.41	4.0
Beverages	Sweetened beverages	Fruit Drinks	757	17.04±0.63	3.8
Grains	Cooked grains	Pasta, noodles, cooked grains	358	35.95±1.00	3.9
		**Colombia (n=1230)**			
Fruits	Fruits	Banana	1421	39.13±0.91	7.1
Grains	Cooked grains	Rice	3214	15.96±0.15	6.6
Grains	Plant-based Protein Foods	Beans, peas, legumes	865	53.78±1.77	5.9
		Fruit drinks	1116	36.74±1.11	5.3
Beverages	Coffee	Coffee	2523	15.18±0.53	4.9
		**Costa Rica (n=798)**			
Protein Foods	Plant-based Protein Foods	Beans, peas, legumes	1213	57.50±1.22	17.6
Grains	Cooked grains	Rice	1845	21.89±0.28	10.2
Mixed Dishes	Mixed Dishes - Mexican	Other Mexican mixed dishes	498	64.78±1.69	8.1
Beverages	Coffee	Coffee	1842	9.67±0.17	4.5

Grains	Bread, Rolls and Tortillas	Yeast breads	1138	11.76±0.35	4.2
**Ecuador (n=800)**					
Mixed Dishes	Mixed Dishes - Soups	Soups	1459	47.72±0.78	13.3
Grains	Cooked grains	Rice	2408	20.22±0.25	10.6
Fruits	Fruits	Banana	583	54.95±2.52	7.0
Mixed Dishes	Mixed Dishes – M/P/S	Meat Mixed Dishes	362	82.05±2.49	5.9
Grains	Bread, Rolls and Tortillas	Rolls and Buns	776	22.98±1.19	3.9
**Peru (n=1113)**					
Grains	Cooked grains	Rice	2574	29.29±0.31	11.7
Grains	Bread, Rolls and Tortillas	Yeast Breads	2482	17.24±0.24	6.6
Protein Foods	Poultry	Chicken, whole pieces	2008	18.70±0.26	5.8
Fruits	Fruits	Banana	853	37.22±0.90	4.9
Mixed Dishes	Mixed Dishes – Grain-based	Rice Mixed Dishes	541	62.39±1.49	4.8
**Venezuela (n=1132)**					
Grains	Quick Breads and Bread Products	Pancakes, waffles, French Toast	2134	43.88±0.50	16.2
Beverages	Coffee	Coffee	1821	23.94±0.31	7.6
Mixed Dishes	Mixed Dishes – Grain-based	Turnovers and other grain-based items	546	60.34±1.83	5.7
Fruits	Fruits	Banana	846	33.80±0.82	5.0
Milk and Dairy	Cheese	Cheese	1932	12.11±0.16	4.1

Note: M/P/S: Meat/Poultry/Seafood; SE: Standard Error.

Supplement table 9 – Food sources of potassium (mg/day) intake among Latin American adolescents and adults. The Latin American Study of Nutrition and Health.

Main group	Subgroup	Category	Cons	M± SE	%
**Argentina (n=1266)**					
Mixed Dishes	Mixed Dishes – M/P/S	Meat Mixed Dishes	781	407.83±8.47	6.2
Mixed Dishes	Mixed Dishes – Grain-based	Pasta Mixed Dishes	794	373.53±11.89	5.9
Beverages	Coffee and tea	Tea	3897	66.98±0.74	5.7
Protein Foods	Meats	Beef, excludes ground	728	354.07±9.14	5.5
Grains	Bread, rolls, tortillas	Yeast Breads	2583	96.21±1.38	5.4
**Brazil (n=2000)**					

Protein Foods	Plant-based protein foods	Beans, Peas, Legumes	3701	283.64±3.19	13.2
Protein Foods	Meats	Beef, excludes ground	1882	383.81±6.02	9.1
Milk and Dairy	Milk	Milk, whole	2382	227.20±3.21	6.8
Protein Foods	Poultry	Chicken, whole pieces	1836	197.06±3.77	4.5
Fruits	Fruits	Other Fruit and Fruit Salads	1056	299.81±9.48	3.9
**Chile (n=879)**					
Fruits	Fruits	Other fruit and fruit salads	751	311.90±7.40	7.1
Grains	Bread, rolls, tortillas	Yeast breads	1880	116.87±1.40	6.7
Vegetables	White potatoes	White potatoes, baked or boiled	281	695.89±32.23	5.9
Beverages	Sweetened beverages	Fruit drinks	757	254.98±9.92	5.8
Vegetables	Vegetables, excluding potatoes	Tomatoes	768	209.09±3.97	4.9
**Colombia (n=1230)**					
Fruits	Fruits	Banana	1421	561.17±13.26	9.7
Vegetables	White potatoes	White Potatoes, baked or boiled	939	577.32±16.49	9.6
Mixed Dishes	Mixed Dishes – M/P/S	Meat Mixed Dishes	493	1076.12±40.90	6.4
Mixed Dishes	Mixed Dishes - Sandwiches	Other Sandwiches	902	519.68±15.16	5.5
Protein Foods	Plant-based protein foods	Beans, peas, legumes	865	511.88±17.48	5.4
**Costa Rica (n=798)**					
Protein Foods	Plant-based protein foods	Beans, peas, legumes	1213	355.16±6.98	12.4
Beverages	Coffee and tea	Coffee	1842	149.39±1.61	7.9
Fruits	Fruits	Banana	532	352.42±13.40	5.4
Grains	Cooked cereals	Rice	1845	86.15±1.12	4.6
Mixed Dishes	Mixed Dishes - Mexican	Other Mexican Mixed Dishes	498	308.60±8.97	4.4
**Ecuador (n=800)**					
Fruits	Fruits	Banana	583	781.26±36.20	10.1
Mixed Dishes	Mixed Dishes – M/P/S	Meat Mixed Dishes	362	1172.18±37.09	8.6
Beverages	100% juices	Citrus Juice	763	289.66±13.09	4.7
Mixed Dishes	Mixed Dishes – M/P/S	Poultry Mixed Dishes	436	450.13±7.50	4.2
Fruits	Fruits	Other Fruit and Fruit Salads	640	285.20±13.91	4.0
**Peru (n=1113)**					
Vegetables	White potatoes	White Potatoes, baked or boiled	1079	418.23±10.64	8.2
Fruits	Fruits	Banana	853	513.76±12.86	8.1
Protein Foods	Poultry	Chicken, whole pieces	2008	173.69±2.38	6.4

Fruits	Fruits	Other fruit and fruit salads	991	264.27±8.68	4.7
Mixed Dishes	Mixed Dishes - Soups	Soups	1214	231.35±5.98	4.3
		**Venezuela (n=1132)**			
Fruits	Fruits	Banana	846	483.93±11.64	9.0
Beverages	100% juices	Other Fruit Juice	1013	314.92±5.79	6.4
Grains	Quick breads and other bread products	Pancakes, waffles, French Toast	2134	131.34±1.96	6.1
Mixed Dishes	Mixed Dishes – M/P/S	Meat Mixed Dishes	507	573.84±27.29	6.0
Beverages	100% juices	Citrus Juice	402	557.55±20.56	4.9
Note: M/P/S: Meat/Poultry/Seafood; SE: Standard Error					

Supplement table 10 – Food sources of fiber (mg/day) intake among Latin American adolescents and adults. The Latin American Study of Nutrition and Health.

Main group	Subgroup	Category	Cons	M± SE	%
		**Argentina (n=1266)**			
Beverages	Coffee and Tea	Tea	3897	8.70±0.13	53.9
Grains	Bread, rolls, tortillas	Yeast breads	2583	1.95±0.03	8.0
Fruits	Fruits	Other fruit and Fruit salads	554	3.70±0.12	3.3
Mixed Dishes	Mixed Dishes – Grain-based	Pasta mixed dishes	794	2.28±0.06	2.6
Mixed Dishes	Mixed Dishes – Pizza	Pizza	372	4.43±0.15	2.5
		**Brazil (n=2000)**			
Protein foods	Plant-based protein foods	Beans, peas, legumes	3701	3.60±0.04	24.1
Grains	Bread, rolls, tortillas	Yeast breads	3465	1.68±0.02	10.5
Fruits	Fruits	Other fruit and fruit salads	1056	3.78±0.12	7.2
Beverages	Coffee and Tea	Coffee	4135	0.64±0.01	4.8
Beverages	100% juices	Other fruit juice	839	2.62±0.08	3.9
		**Chile (n=879)**			
Grains	Bread, rolls, tortillas	Yeast Breads	1880	2.29±0.03	17.4
Fruits	Fruits	Other fruit and fruit salads	751	4.17±0.09	12.7
Protein foods	Seafoods	Fish	1029	1.64±0.04	6.9
Protein foods	Plant-based protein foods	Bean, pea, legume dishes	115	10.82±0.93	5.0
Vegetables	White Potatoes	White potatoes, baked or boiled	281	4.14±0.20	4.7

**Colombia (n=1230)**					
Protein foods	Plant-based protein foods	Beans, peas, legumes	865	8.23±0.27	14.2
Fruits	Fruits	Banana	1421	2.99±0.07	8.5
Grains	Bread, rolls, tortillas	Yeast breads	1418	2.24±0.07	6.4
Fruits	Fruits	Other fruit and fruit salads	697	4.26±0.21	5.9
Vegetables	White Potatoes	White potatoes, baked or boiled	939	3.04±0.08	5.7
**Costa Rica (n=798)**					
Protein foods	Plant-based protein foods	Beans, peas, legumes	1213	7.51±0.15	28.2
Mixed Dishes	Mixed Dishes – Mexican	Other Mexican Mixed dishes	498	6.39±0.18	9.9
Beverages	Coffee and Tea	Coffee	1842	1.39±0.01	7.9
Grains	Bread, rolls, tortillas	Yeast breads	1138	1.63±0.04	5.8
Fruits	Fruits	Other fruit and fruit salads	490	3.25±0.16	4.9
**Ecuador (n=800)**					
Mixed Dishes	Mixed Dishes – Soups	Soups	1459	5.13±0.12	22.0
Fruits	Fruits	Banana	583	4.28±0.19	8.4
Mixed Dishes	Mixed Dishes – M/P/S	Meat Mixed Dishes	362	6.67±0.22	7.5
Fruits	Fruits	Other fruit and fruit salads	640	3.43±0.16	7.4
Grains	Cooked Grains	Rice	2408	0.58±0.01	4.7
**Peru (n=1113)**					
Grains	Bread, rolls, tortillas	Yeast Breads	2482	1.53±0.02	8.8
Mixed Dishes	Mixed Dishes - Soups	Soups	1214	3.55±0.09	8.4
Fruits	Fruits	Other fruit and fruit salads	991	3.72±0.11	8.3
Protein foods	Plant-based protein foods	Beans, peas, legumes	643	5.49±0.19	8.1
Vegetables	White Potatoes	White Potatoes, baked or boiled	1079	2.60±0.07	6.5
**Venezuela (n=1132)**					
Grains	Quick breads, and bread products	Pancakes, waffles, French Toast	2134	2.95±0.03	17.7
Beverages	100% juices	Other fruit juice	1013	3.46±0.06	9.1
Mixed Dishes	Mixed Dishes - Soups	Soups	398	8.02±0.40	7.8
Fruits	Fruits	Banana	846	2.57±0.07	6.2
Grains	Bread, rolls, tortillas	Yeast breads	958	2.07±0.04	5.6

Note: M/P/S: Meat/Poultry/Seafoods; SE: Standard Error

Supplement table 11– Food sources of iron (mg/day) intake among Latin American adolescents and adults. The Latin American Study of Nutrition and Health.

Main group	Subgroup	Category	Cons	M± SE	%
		**Argentina (n=1266)**			
Grains	Bread, Rolls, Tortillas	Yeast Breads	2583	2.56±0.03	17.6
Mixed Dishes	Mixed Dishes – M/P/S	Meat Mixed Dishes	781	4.74±0.11	8.9
Mixed Dishes	Mixed Dishes – Grain-based	Pasta Mixed Dishes	794	3.93±0.10	7.6
Beverages	Tea	Tea	3897	0.71±0.01	7.4
Mixed Dishes	Mixed Dishes – Pizza	Pizza	372	5.45±0.19	5.3
		**Brazil (n=2000)**			
Grains	Bread, Rolls, Tortillas	Yeast Breads	3465	2.35±0.02	16.2
Protein Foods	Plant-based Protein Foods	Beans, Peas, Legumes	3701	1.87±0.02	13.7
Grains	Cooked grains	Rice	4680	1.23±0.01	11.4
Protein Foods	Meats	Beef, excludes ground	1882	2.50±0.04	9.4
Protein Foods	Poultry	Chicken, whole pieces	1836	0.98±0.02	3.6
		**Chile (n=879)**			
Grains	Bread, Rolls, Tortillas	Yeast Breads	1880	3.18±0.04	27.2
Protein Foods	Seafoods	Fish	1029	2.64±0.06	12.4
Grains	Cooked grains	Pasta, noodles, cooked grains	358	2.52±0.07	4.1
Grains	Cooked grains	Rice	524	1.64±0.03	3.9
Protein Foods	Meats	Beef, excludes ground	393	1.85±0.07	3.3
		**Colombia (n=1230)**			
Grains	Cooked grains	Rice	3214	1.54±0.01	12.1
Grains	Bread, Rolls, Tortillas	Yeast breads	1418	2.65±0.06	9.2
Protein Foods	Plant-based Protein Foods	Beans, peas, legumes	865	3.61±0.12	7.6
Mixed Dishes	Mixed Dishes – M/P/S	Meat Mixed Dishes	493	4.01±0.14	4.8
Protein Foods	Meats	Beef, excludes ground	1022	1.74±0.03	4.3
		**Costa Rica (n=798)**			
Grains	Cooked grains	Rice	1845	2.06±0.03	16.9
Protein Foods	Plant-based Protein Foods	Beans, peas, legumes	1213	2.07±0.04	11.2
Grains	Bread, Rolls, Tortillas	Yeast Breads	1138	2.16±0.04	10.9
Mixed Dishes	Mixed Dishes – Mexican	Other Mexican Mixed Dishes	498	3.05±0.08	6.8

Protein Foods	Poultry	Chicken, whole pieces	441	1.82±0.09	3.6
**Ecuador (n=800)**					
Grains	Cooked grains	Rice	2408	2.01±0.02	20.3
Mixed Dishes	Mixed Dishes – Soups	Soups	1459	2.73±0.05	14.6
Grains	Bread, Rolls, Tortillas	Rolls and buns	776	2.71±0.07	8.8
Mixed Dishes	Mixed Dishes – M/P/S	Meat Mixed Dishes	362	3.9±0.13	5.5
Mixed Dishes	Mixed Dishes – M/P/S	Poultry Mixed Dishes	436	2.47±0.05	4.30
**Peru (n=1113)**					
Grains	Cooked grains	Rice	2574	2.89±0.03	21.2
Grains	Bread, Rolls, Tortillas	Yeast Breads	2482	2.02±0.02	14.1
Mixed Dishes	Mixed Dishes – Grain-based	Rice Mixed Dishes	541	5.12±0.12	7.2
Protein Foods	Poultry	Chicken, whole pieces	2008	0.86±0.01	4.9
Mixed Dishes	Mixed Dishes – Soups	Soups	1214	1.48±0.03	4.3
**Venezuela (n=1132)**					
Grains	Quick bread and bread products	Biscuits, muffins, quick breads	2134	3.48±0.04	23.7
Grains	Bread, Rolls, Tortillas	Yeast breads	958	2.97±0.05	9.2
Grains	Cooked Grains	Rice	1482	1.35±0.02	6.2
Mixed Dishes	Mixed Dishes – Grain-based	Turnovers and other grain-based items	546	3.09±0.09	5.4
Mixed Dishes	Mixed Dishes – M/P/S	Meat Mixed Dishes	507	3.17±0.07	4.5

Note: M/P/S: Meat/Poultry/Seafood; SE: Standard Error
